# Evaluation of the Survivability of Microorganisms Deposited on Filtering Respiratory Protective Devices under Varying Conditions of Humidity

**DOI:** 10.3390/ijerph13010098

**Published:** 2016-01-04

**Authors:** Katarzyna Majchrzycka, Małgorzata Okrasa, Justyna Skóra, Beata Gutarowska

**Affiliations:** 1 Department of Personal Protective Equipment, Central Institute for Labour Protection—National Research Institute, Łódź 90-133, Poland; maokr@ciop.lodz.pl; 2 Institute of Fermentation Technology and Microbiology, Lodz University of Technology, Łódź 90-924, Poland; justyna-skora@wp.pl (J.S.); beata.gutarowska@p.lodz.pl (B.G.)

**Keywords:** filtering respiratory protective devices, FRPDs, microorganisms, filter material, humidity conditions

## Abstract

Bioaerosols are common biological factors in work environments, which require routine use of filtering respiratory protective devices (FRPDs). Currently, no studies link humidity changes in the filter materials of such devices, during use, with microorganism survivability. Our aim was to determine the microclimate inside FRPDs, by simulating breathing, and to evaluate microorganism survivability under varying humidity conditions. Breathing was simulated using commercial filtering facepiece respirators in a model system. Polypropylene melt-blown nonwoven fabrics with moisture contents of 40%, 80%, and 200%, were used for assessment of microorganisms survivability. A modified AATCC 100-2004 method was used to measure the survivability of ATCC and NCAIM microorganisms: *Escherichia coli*, *Staphylococcus aureus*, *Bacillus subtilis*, *Candida albicans* and *Aspergillus niger*. During simulation relative humidity under the facepiece increased after 7 min of usage to 84%–92% and temperature increased to 29–30 °C. *S. aureus* survived the best on filter materials with 40%–200% moisture content. A decrease in survivability was observed for *E. coli* and *C. albicans* when mass humidity decreased. We found that *B. subtilis* and *A. niger* proliferated for 48–72 h of incubation and then died regardless of the moisture content. In conclusion, our tests showed that the survivability of microorganisms on filter materials depends on the amount of accumulated moisture and microorganism type.

## 1. Introduction

Biological factors constitute a very important problem of occupational medicine and public health. The threat posed by biological agents is increasingly seen as a global issue, mainly due to the rapid spread of microorganisms over time, and across work and living environments due to modern pace of work and life. It is estimated that, at least, several hundred million people worldwide are exposed to these factors in processes related to their work environment [1,2]. This problem is particularly acute in tropical and subtropical countries, where people often come in contact with numerous parasites and inhale harmful bioaerosols [3,4,5]. In the temperate zone, a significant number of cases of diseases caused by biological factors is observed, not only among health professionals [6] and dentists [7], but also people employed in agricultural production [8,9,10,11,12,13,14], wood industry [15,16], composting plants [17,18,19], sewage and solid waste treatment plants [20,21], and the dairy industry [22,23]. 

In the work environment, biological agents occur most often in the form of bioaerosols. Therefore, it is common to use personal protective equipment (PPE), particularly, disposable and reusable filtering respiratory protective devices (FRPDs). The lack of adequate protection of the respiratory system in workers exposed to biological agents can lead to asthma, allergic alveolitis, organic dust toxic syndrome (ODTS), byssinosis, chronic bronchitis, allergic rhinitis, irritation of mucous membranes, sick building syndrome (SBS), and certain infectious diseases and cancer [1,24,25,26].

There are many theories and studies on the selection of FRPDs in relation to biological hazards [27,28,29]. In addition to FRPDs (respirators), surgical masks are often used in the work environment. According to EU regulations, these belong to the group of medical devices whose primary function is to control infections. They are designed to prevent the spread of infection from the wearer’s exhaled breath to potentially susceptible persons [30]. These masks are not a good means of protecting workers from exposure to biological agents, especially in the work environment with a high concentration of organic dust including microorganisms.

Previous studies have tested the survivability of microorganisms on filter materials, which constitute the basic structural material of FRPDs [31,32,33,34,35,36,37,38,39]. The growth of microorganisms on filter materials is mainly influenced by the availability of organic matter, humidity and temperature, the addition of biocide and the type of microorganism [40,41]. The results have outlined a new direction for the development of protective properties of FRPDs against bioaerosols, *i.e.*, biocidal properties of filter materials for the construction of reusable FRPDs. The materials are designed to protect against proliferation of microorganisms, deposited on them during FRPD use, so that the respirator itself is not a source of infection for the user. Furthermore, it is important to carry out studies on the survivability of microorganisms on filter materials under conditions similar to those prevailing in the work environment where FRPDs are typically used. This makes it possible to adequately evaluate the extent to which FRPDs protect against bioaerosols in the “worst-case” scenario.

Currently, no studies link humidity changes occurring in filter materials during FRPD use with the survivability of the deposited microorganisms. Therefore, the aim of this study was to determine the microclimate inside the FFRs during simulated breathing, and to evaluate the survivability of different types of microorganisms deposited on the filter materials of the FRPDs under varying humidity conditions.

## 2. Experimental Section

### 2.1. Tested Respirators and Filter Materials

The level of humidity in the FRPDs during breathing cycles was measured using commercial reusable FFRs, consistent with the requirements of a harmonized standard [42] under EU directive 89/686/EEC [43]. In terms of safety parameters, these were filtering facepieces of protection class III (FF P3). The filtration efficiency of such equipment relative to aerosol particles (NaCl) and fog paraffinic oil is 99% at the volume flow rate of 95 L/min. The FFRs were designed to be reusable (symbol “R” according to EN 149) against any aerosols, in accordance with the requirements of the standards applicable to the common EU market. The FFRs consisted of six layers of filter materials. All filter materials were made of polypropylene polymer and produced using two different manufacturing procedures. Inner and outer layers of the FFRs were made of thin spun-bonded nonwoven fabric. The basic filtering layers, responsible for filtration efficiency, were produced using the melt-blown technology. This ensured thin fibres, which directly affected the filtration efficiency against harmful particles of aerosols. The two outer layers were made of nonwovens with surface weights of 40 g/m^2^ ± 5% and 80 g/m^2^ ± 5%, the three middle layers of nonwoven fabric had a total surface weight of 90 g/m^2^ ± 15% and the inner layer of nonwoven had a surface weight of 17 g/m^2^ ± 5%.

To test the survivability of microorganisms in the FRPDs, polypropylene melt-blown nonwoven fabrics were used, as they were considered responsible for catching harmful microorganisms from a stream of contaminated air. Commercially-produced fabrics were used to construct the above FFRs. On average, the surface weight of each nonwoven fabric sample was 30 g/m^3^ ± 10%. 

### 2.2. Experimental Design and Test Conditions

#### Microclimate inside the FFRs

**Figure 1:**
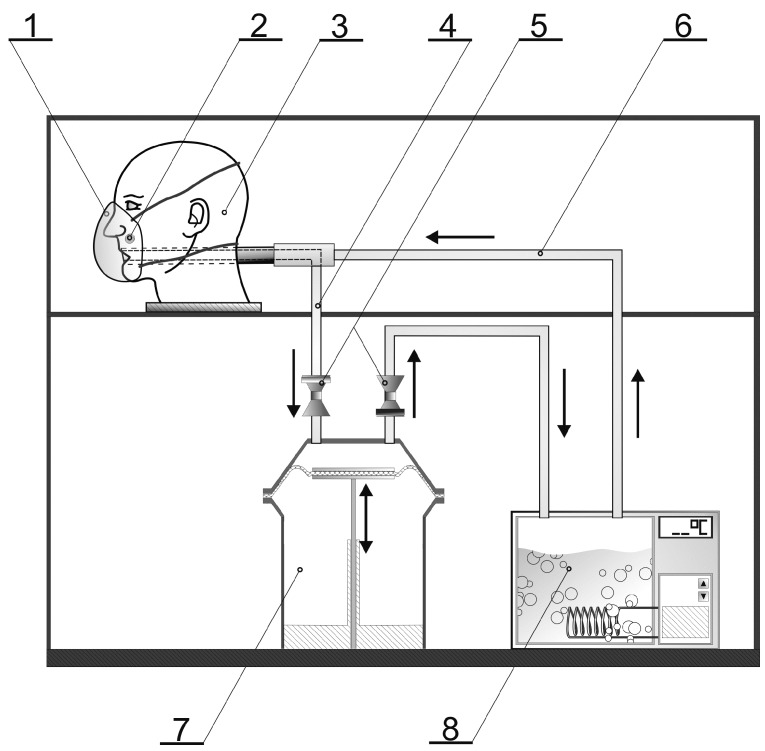
The scheme of the test stand used to simulate breathing cycles while wearing an FFR (1—FFR, 2—integrated temperature and humidity data logger, 3—Sheffield dummy head, 4—inhalation line, 5—valves, 6—exhalation line, 7—breathing machine, 8—saturator).

The test stand used to simulate breathing in our experiments is shown in Figure 1.

The test stand consisted of a breathing machine, a Sheffield dummy head on which an FFR was mounted, and a saturator located in the exhalation line between the dummy head and the breathing machine. The breathing machine was set at 25 cycles/min and 2 L/stroke, which corresponded to 25 inhalations and 25 exhalations per minute with respiratory volume of 2 liters. The airflow pattern of the breathing machine was sinusoidal. The saturator was set at a temperature exceeding 37 °C, so that the temperature of the air exhaled from the dummy head’s mouth, after passing through the system, was 37 ± 2 °C. The relative humidity and temperature of the environment was monitored in the respiratory space between the dummy head and the tested FFRs. Integrated temperature and humidity data logger 1-Wire® Hygrochron™ (type DS1923, Maxim Integrated) was used. The temperature and relative humidity of the environment were 23–25 °C and 47%–55%, respectively.

The study was conducted according to the following procedure: an FFR was placed on the dummy head, the simulation of breathing was carried out for 3.5 h, the FFR was taken off the dummy head, a one-hour break in the use of FFR was simulated (e.g., a lunch break), the FFR was again placed on the dummy head, the simulation of breathing was carried out for another 3.5 h, and the FFR was taken off the dummy head. Two series of simulations were performed. Each series was conducted using a new FFR. The accuracy of fit was based on the determination of a nominal protection factor, *i.e.*, potential maximum protection factor calculated using the maximum percentage inward leakage permitted for FFRs of third protection class according to European standards [42]. However, it is worth mentioning that this level of protection is unlikely to be achieved in real conditions of use of FFRs.

### 2.3. Microorganisms

The study involved testing five strains from pure culture collections. Four were obtained from the American Type Culture Collection (ATCC), *Escherichia coli* 10536, *Staphylococcus aureus* 6538, *Candida albicans* 10231, *Aspergillus niger* 16404, and one, *Bacillus subtilis* 01644, was obtained from the National Collection of Agricultural and Industrial Microorganisms (NCAIM). The strains were selected based on their taxonomic classification (*i.e.*, Gram-positive cocci, Gram-negative rods, Gram-positive bacilli, yeast, and mold). The strains were also characterized by their varying survivability in the environment *i.e.*, whether they produced endospores (*B. subtilis*), spores (*A. niger*), or just vegetative cells (*E. coli*, *S. aureus*, *C. albicans*). Moreover, according to Directive 2000/54/ EC [44] and the Classification of Harmful Biological Factors (developed by the Institute of Rural Health in Lublin) [45], the species selected for testing belong to Risk Group II and may pose a potential threat to humans in the work environment.

The microorganisms were stored on agar slants: bacteria on TSA (Tryptic Soy Agar, Merck, Germany), and molds and yeasts on MEA (Malt Extract Agar, Merck, Germany) at a temperature of 4 °C. Bacteria were activated in TSB medium (Tryptic Soy Broth, Merck, Germany) and fungi in MEB medium (Malt Extract Broth, Merck, Germany) at a temperature of 30 °C for 24 h (bacteria), 27 °C for 24 h (yeasts) and 72 h (moulds).

A microorganism inoculum was prepared. Bacteria and yeast colonies were transferred into 10 mL of TSB and MEB medium, respectively, and incubated in conditions as described above. In the case of mold, colonies from MEA slants were washed using 10 mL distilled water with 0.01% of Tween^®^80. The number of microorganisms in the inoculum was determined using the plate count method and a Thom chamber. The average density of the suspension of microorganisms ranged from 1.3 × 10^8^ to 1.3 × 10^10^ cfu/mL (bacteria) or from 3.9 × 10^7^ to 6.5 × 10^7^ cfu/mL (fungi). Verifying the density of the inoculum in the Thoma cell counting chamber, in the inoculum approximately 20% of all microorganism cells constituted to *B. subtilis* spores and 99% to *A. niger* spores. Removal efficiency of the tested microorganisms was 52.2% ± 4.1%.

### 2.4. Survivability of Microorganisms on Filter Materials

The survivability of microorganisms on filter materials was measured using a modified quantitative method, AATCC 100-2004 [46] with the following modification: the maximum incubation time following the estimated time of use for reusable FFR was 120 h.

The tests were conducted for samples of filter materials with a surface area of 4 cm^2^ each. Three levels of the mass humidity of the materials (40%, 80%, and 200%) were determined. Humidity levels of 40% and 80% were determined according to the results of the breathing simulation and corresponded to the estimated humidity of the filter material during a break in the usage of respirators and during breathing with a half mask. Humidity 200% was chosen as a model of very high humidity of the filter material (worst-case scenario). Three levels of the mass humidity of the materials were determined by applying sterile distilled water onto the samples. Sterile distilled water was applied homogenously onto the samples using a pipette with sterile tips for the distribution of small water droplets over the whole sample. Mass humidity (H), defined as the amount of moisture accumulated in the filter material, was determined by a gravimetric method (Radwag moisture analyzer MA 50/1.R, Poland) and calculated using the formula:

(1) $$H=\frac{m_{H_{2}O}}{m_{m}}\times 100\%$$

$m_{H_{2}O}$: the mass of water (g); $m_{m}$: the mass of the filter material with an area of 4 cm^2^ (average mass *m_m_* = 0.0125 g).

Next, 10 µL of standardized inoculum of microorganisms was applied evenly in the same way using pipette with sterile tips. Filter material samples were prepared in two independent repetitions of the experiment and placed in a climatic chamber (Binder, Germany) at 28 °C and relative humidity RH of 80%. The samples were collected at 0 (immediately after inoculum application) 8, 24, 48, 72, and 120 h.

The samples were then placed in 50 mL of sterile saline (0.85% NaCl) and shaken for 10 min (150 rev/min, shaker Elpin+, Poland) to wash out the microorganisms from the tested materials. Bacteria were incubated at 30 °C for 24–48 h on TSA medium and fungi on MEA medium at 27 °C for 3–5 days. The number of microorganisms (cfu/sample) was determined in two independent experiments using the plate count method (dilutions from 10^0^ to 10^5^ in three repetitions). This resulted in a 30 repetitions for one microorganism, for each humidity level and for each incubation time.

The survival rate of microorganisms on the filter material after different incubation periods (S) was determined using the formula:

(2) $$S=\frac{N_{t}}{N_{0}}\times 100\%$$

*N*_0_, *N_t_*: the number of microorganisms present on the filter material (cfu/sample); *N*_0_: at time t = 0 h; *N_t_*: after a certain period of incubation (t = 8, 24, 48, 72, 120 h).

### 2.5. Statistical Analysis

The arithmetic mean and standard deviation for the number of microorganisms on the surface of the tested materials were calculated. Differences between the number of microorganisms after a particular incubation time and the control samples were analysed using one-way analysis of variance (ANOVA). Differences were considered significant at *p* < 0.05. All data were analyzed using the statistical software program Origin 6.1.

## 3. Results and Discussion

### 3.1. Microclimate inside the FFRs

**Figure 2:**
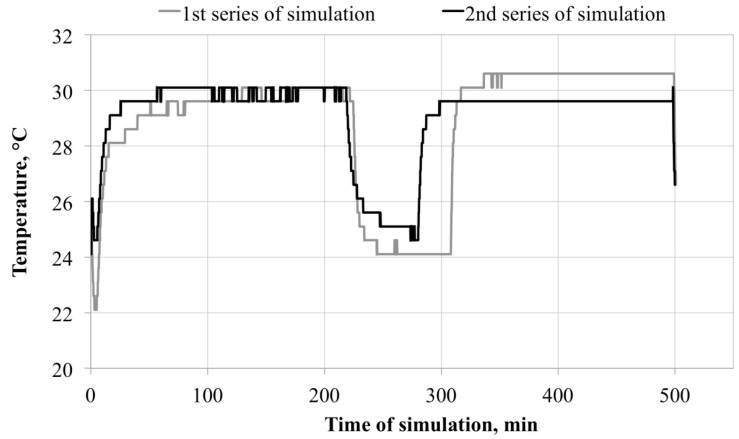
Temperature changes inside the FFRs during simulated breathing cycles.

The results of temperature measurements performed directly under the facepiece are shown in Figure 2. 

In the first and second series of simulated breathing cycles, a microclimate with similar temperature was created inside the FFRs. After one hour of breathing, the temperature in the FFR under the facepiece reached 29–30 °C. Differences between the two series were not statistically significant. The temperature inside the FFR was stable and lower than the normal body temperature of a healthy worker. The microclimate temperature inside the FFR was only slightly affected by breaks in the use of the equipment.

**Figure 3:**
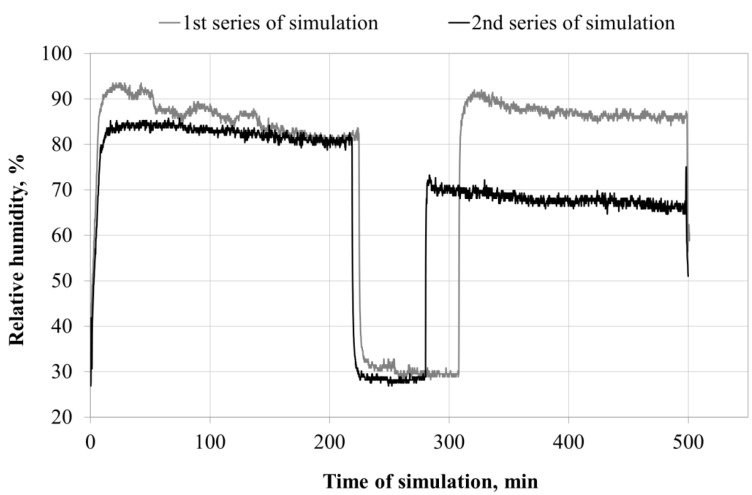
Changes in relative humidity inside the FFR during simulated breathing cycles.

The values of relative humidity in the space directly under the facepiece are shown in Figure 3.

The values of relative humidity inside the FFR microclimate obtained during the two series of simulations show that the start of simulation is followed by an immediate sharp increase in relative humidity up to 84%–92% (first and second series) inside the respirator. The relative humidity within the entire simulated breathing time ranged from 80% to 92%. When the simulation of breathing was started again after a one-hour break, the relative humidity inside the FFR increased again to 91% (first series) and 69% (second series). 

**Figure 4:**
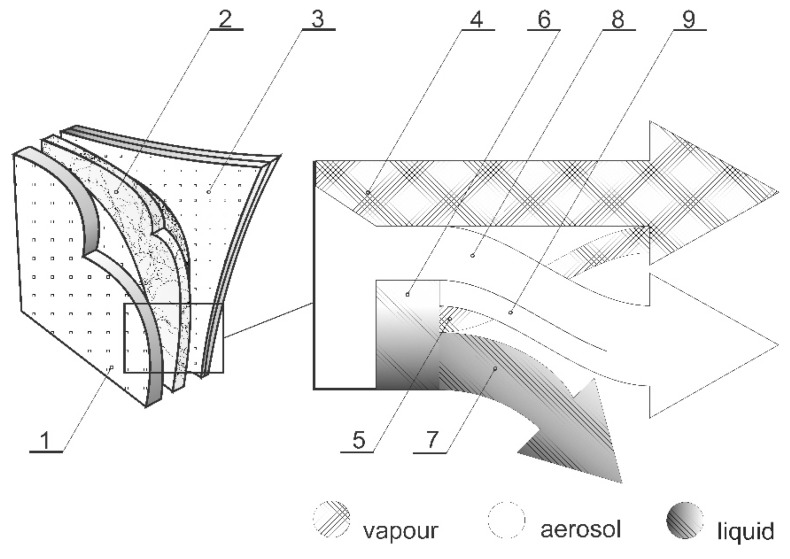
Moisture distribution in the FFR during its use (1—inner layer, 2—filtering layer, 3—outer layers, 4—vapor penetration, 5—evaporation, 6—collected water, 7—drained water, 8—penetration of droplets, 9—re-entrainment). Adapted from Raynor and Leith [47].

The difference in relative humidity changes inside the FFR (22%) between both series may result from the course of moisture condensation on the surface of the filter materials, and the rate of migration of the collected liquid to various structural layers of the FFR. This can be attributed to differences in the production of particular FFRs and in head adjustments in the successive phases of series I and II. The phenomena accompanying moisture deposition on filter materials were described by Raynor and Leith [47]. They found that the behavior of a liquid during deposition on filter materials is more diverse than that of particulate matter. Agglomerates of accumulated moisture move, dry up, and break off (Figure 4). After merging into one whole, droplets smoothly flow down the filter materials. The amount of the collected condensing liquid depends on the geometry of the filter, filtration efficiency and the surface tension of the retained liquid relative to the force of gravity. The collected water is drained from the fibers as soon as the minimum saturation is reached. It was observed that the air that passes during the breathing phase can cause migration of accumulated moisture into the filtering devices, and the air stream that comes from the moistened filter may be saturated with water vapor.

The amount of water accumulated on the filter materials has a strong impact on the pressure drop on these materials (breathing resistance) [48,49,50]. There is a slight increase in the pressure drop in the initial breathing phase. It then increases exponentially due to the accumulation of liquid on the surface of the filter materials and the formation of menisci between fibers. Eventually, the pressure drop reaches a constant level and a balance between the process of moisture retention and evaporation is created. These changes could affect the breathing resistance during the simulation of breathing in the FFRs, thus causing differences in the ability to adapt the FFRs to the dummy head model.

Both, literature data and results of the breathing simulations in the FFR confirm the fact that the conditions prevailing inside the respirators are favorable for the continuous gathering of moisture from the exhaled air. This study shows that it is maintained as long as the FFR is used even if its operation is interrupted. These conditions may affect the survivability of microorganisms deposited on the filter materials during the use of FFR at the workplace and during breaks.

### 3.2. Survivability of Microorganisms on Filter Materials

Table 1 shows the number of microbial cells present on the filter materials after incubation at various levels of mass humidity. It is assumed that the set mass humidity values of 40%, 80%, and 200% are disadvantageous, suboptimal, and optimal conditions, respectively, for the growth of microorganisms.

**Table 1:** The number of microorganisms on filter materials after different time intervals.

Microorganism	Mass Humidity, %	The Number of Cells on the Nonwoven Fabric after Incubation, cfu/sample (*n* = 6)					
		0 h	8 h	24 h	48 h	72 h	120 h
***Escherichia coli***	**40**	M: 8.75 × 10^5^	M: 9.53 × 10^5^	M: 1.79 × 10^4^ *	M: 9.18 × 10^1^ *	M: 2.07 × 10^1^ *	M: 9.83 × 10^0^ *
		SD: 1.42 × 10^5^	SD: 1.14 × 10^5^	SD: 4.05 × 10^3^	SD: 3.82 × 10^1^	SD: 1.18 × 10^1^	SD: 6.91 × 10^0^
	**80**	M: 1.76 × 10^6^	M: 2.77 × 10^6^ *	M: 4.45 × 10^5^ *	M: 1.93 × 10^5^ *	M: 1.11 × 10^4^ *	M: 2.75 × 10^2^ *
		SD: 7.34 × 10^5^	SD: 3.95 × 10^5^	SD: 1.92 × 10^5^	SD: 1.72 × 10^4^	SD: 6.45 × 10^3^	SD: 1.08 × 10^2^
	**200**	M: 2.77 × 10^6^	M: 2.82 × 10^6^	M: 2.21 × 10^6^ *	M: 1.82 × 10^6^ *	M: 1.62 × 10^6^ *	M: 2.50 × 10^3^ *
		SD: 2.73 × 10^5^	SD: 9.81 × 10^5^	SD: 3.93 × 10^5^	SD: 6.17 × 10^5^	SD: 1.83 × 10^5^	SD: 7.90 × 10^2^
***Staphylococcus aureus***	**40**	M: 1.93 × 10^4^ *	M: 3.23 × 10^4^ *	M: 4.78 × 10^4^ *	M: 5.95 × 10^4^ *	M: 1.14 × 10^5^	M: 4.02 × 10^5^ *
		SD: 8.91 × 10^3^	SD: 4.08 × 10^3^	SD: 1.87 × 10^4^	SD: 2.08 × 10^4^	SD: 2.08 × 10^5^	SD: 2.19 × 10^5^
	**80**	M: 1.36 × 10^4^	M: 1.74 × 10^5^ *	M: 8.75 × 10^5^ *	M: 1.06 × 10^6^ *	M: 1.27 × 10^6^ *	M: 1.58 × 10^6^ *
		SD: 3.29 × 10^3^	SD: 2.99 × 10^4^	SD: 1.93 × 10^5^	SD: 2.41 × 10^5^	SD: 2.43 × 10^5^	SD: 1.46 × 10^5^
	**200**	M: 1.57 × 10^4^	M: 1.51 × 10^5^ *	M: 1.14 × 10^6^ *	M: 1.34 × 10^6^ *	M: 2.01 × 10^6^ *	M: 2.48 × 10^6^ *
		SD: 1.65 × 10^4^	SD: 3.69 × 10^4^	SD: 1.61 × 10^5^	SD: 1.70 × 10^5^	SD: 3.22 × 10^5^	SD: 4.60 × 10^5^
***Bacillus subtilis***	**40**	M: 4.37 × 10^3^	M: 6.97 × 10^3^ *	M: 1.67 × 10^4^ *	M: 4.38 × 10^4^ *	M: 9.58 × 10^4^ *	M: 7.13 × 10^4^ *
		SD: 1.50 × 10^3^	SD: 1.10 × 10^3^	SD: 8.16 × 10^3^	SD: 1.82 × 10^4^	SD: 2.64 × 10^4^	SD: 1.45 × 10^4^
	**80**	M: 6.70 × 10^3^	M: 1.69 × 10^5^ *	M: 1.98 × 10^5^	M: 2.02 × 10^5^ *	M: 1.61 × 10^5^ *	M: 1.04 × 10^5^ *
		SD: 2.74 × 10^3^	SD: 4.58 × 10^4^	SD: 2.35 × 10^5^	SD: 7.65 × 10^4^	SD: 1.46 × 10^5^	SD: 1.81 × 10^4^
	**200**	M: 1.30 × 10^4^	M: 1.15 × 10^5^ *	M: 2.04 × 10^5^ *	M: 5.37 × 10^5^ *	M: 4.24 × 10^5^ *	M: 5.21 × 10^4^ *
		SD: 5.93 × 10^3^	SD: 1.70 × 10^4^	SD: 6.29 × 10^4^	SD: 2.99 × 10^5^	SD: 1.47 × 10^5^	SD: 3.72 × 10^4^
***Candida albidans***	**40**	M: 3.10 × 10^3^	M: 5.61 × 10^4^	M: 2.65 × 10^4^ *	M: 1.78 × 10^4^ *	M: 1.57 × 10^4^ *	M: 1.50 × 10^4^ *
		SD: 1.33 × 10^3^	SD: 6.20 × 10^4^	SD: 5.89 × 10^3^	SD: 3.29 × 10^3^	SD: 1.66 × 10^3^	SD: 5.42 × 10^3^
	**80**	M: 2.78 × 10^3^	M: 5.03 × 10^4^ *	M: 4.94 × 10^4^ *	M: 4.63 × 10^4^	M: 4.44 × 10^4^ *	M: 3.12 × 10^4^ *
		SD: 1.47 × 10^3^	SD: 8.82 × 10^3^	SD: 1.60 × 10^4^	SD: 5.83 × 10^4^	SD: 1.32 × 10^4^	SD: 1.35 × 10^4^
	**200**	M: 2.48 × 10^3^	M: 5.32 × 10^4^ *	M: 3.81 × 10^4^ *	M: 2.97 × 10^4^ *	M: 2.96 × 10^4^ *	M: 2.96 × 10^4^ *
		SD: 8.68 × 10^2^	SD: 1.97 × 10^4^	SD: 1.52 × 10^4^	SD: 9.94 × 10^3^	SD: 2.21 × 10^4^	SD: 1.84 × 10^4^
***Aspergillus niger***	**40**	M: 1.38 × 10^3^	M: 1.22 × 10^3^	M: 1.27 × 10^3^	M: 1.82 × 10^3^	M: 1.57 × 10^3^	M: 1.10 × 10^3^
		SD: 5.46 × 10^2^	SD: 8.82 × 10^2^	SD: 8.62 × 10^2^	SD: 7.11 × 10^2^	SD: 5.66 × 10^2^	SD: 5.21 × 10^2^
	**80**	M: 2.33 × 10^3^	M: 1.60 × 10^3^	M: 2.08 × 10^3^	M: 2.61 × 10^3^	M: 2.27 × 10^3^	M: 1.08 × 10^3^ *
		SD: 1.06 × 10^3^	SD: 5.69 × 10^2^	SD: 7.55 × 10^2^	SD: 1.78 × 10^3^	SD: 9.84 × 10^2^	SD: 2.14 × 10^2^
	**200**	M: 2.07 × 10^3^	M: 1.72 × 10^3^	M: 2.32 × 10^3^	M: 3.19 × 10^3^	M: 2.65 × 10^3^	M: 1.49 × 10^3^
		SD: 1.02 × 10^3^	SD: 3.92 × 10^2^	SD: 7.55 × 10^2^	SD: 1.90 × 10^3^	SD: 4.64 × 10^2^	SD: 5.04 × 10^2^

Notes: n: number of samples, M: mean, SD: standard deviation, * statistically significant difference between the number of cells on the nonwoven fabric at each incubation time and the time t_0_, based on ANOVA.

The number of microorganisms on the tested nonwoven fabric after eight hours of incubation varied significantly for *S. aureus* and *B. subtilis* under the three mass humidity conditions, for *E. coli* at a mass humidity of 80%, and for *C. albicans* at a mass humidity of 80% and 200%. In the following incubation times, a decrease in the number of *E. coli* cells was observed compared to the number of cells deposited on the material at the time t_0_. The number of cells of the remaining bacteria and yeast increased significantly within 24 h—five days of incubation relative to t_0_. In general there were no statistically significant changes in the numbers of the mold *A. niger* in all tested incubation times and mass humidity conditions.

**Figure 5:**
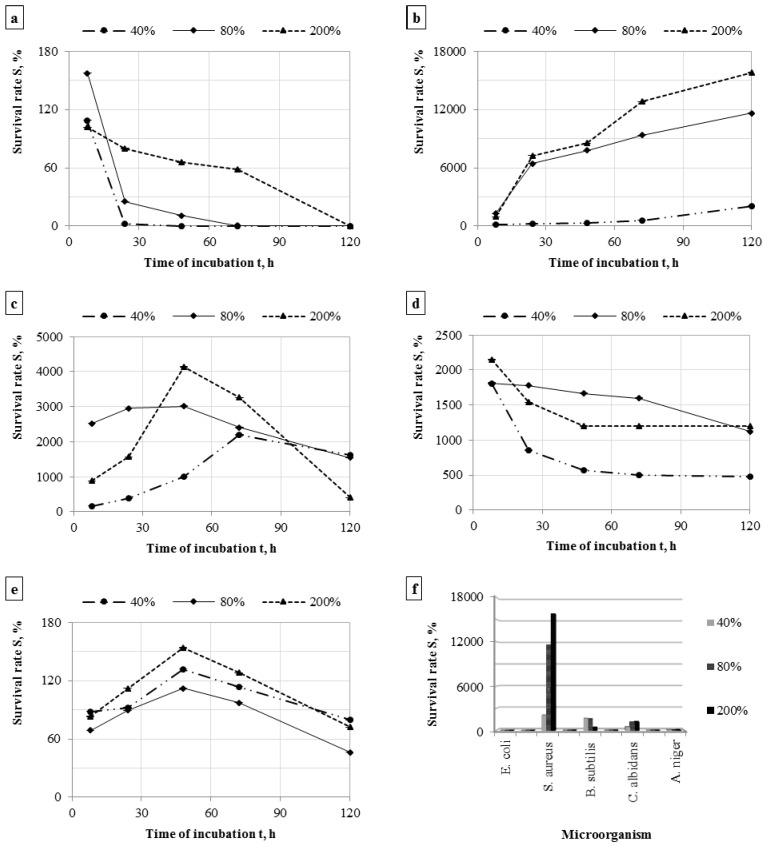
The survival rate of bacteria during incubation on the filter materials, number of samples *n* = 6 (**a**) *Escherichia coli*; (**b**) *Staphylococcus aureus*; (**c**) *Bacillus subtilis*; (**d**) *Candida albidans*; (**e**) *Aspergillus niger*; and (**f**) the survival rate of microorganisms after 120 h of incubation on the filter materials with different moisture content.

The dynamics of changes in the number of microorganisms in subsequent incubation times on the tested filter material is expressed as the survival rate. This parameter depending on the accumulated moisture in the filter materials is shown in Figure 5a–f.

The model study shows that the survivability of microorganisms on the filter materials in the FFRs depends on the amount of moisture accumulated in the filter material and, above all, on the type of microorganism. The best survivability on filter materials with a moisture content of 40%–200% was demonstrated by *Staphylococcus aureus* (Figure 5f). A decrease in survivability on the filter material was observed for yeast *Candida albicans* (Figure 5d) and bacteria *Escherichia coli* (Figure 5a). The polypropylene nonwoven fabric was probably not a conducive environment for proliferation of these species due to their decrease. As for *Bacillus subtilis* and *Aspergillus niger*, it is demonstrated that these microorganisms proliferate on filter materials within 48–72 h of incubation and then probably die (Figure 5c,e).

The results confirm the previous observations of Gutarowska and Michalski [40] and Majchrzycka *et al.* [41]. The authors argue that the survivability of microorganisms on the nonwoven filter can be varied. The type of microorganism, the physiological condition, and the structure of the cell wall have a high impact in this case.

Pasanen *et al.* (1993) argue that storing respirator filters in high humidity conditions results in heavy microbial contamination, particularly when the filter material is biodegradable. Filters exposed to microorganisms present in an agriculture barn and in a wastewater treatment plant were stored at 98% RH for up to 35 days and tested. After the storage, the number of bacteria including actinomycetes was 1–3 orders of magnitude higher, and the concentration of fungi was larger or smaller, depending on the tested respirator filter [51]. In other studies, Pasanen *et al.* (1994) indicate a strong correlation between the visual growth of mold *Stachybotrys chartarum* and cellulose content in filters at RH 100%. It is also shown that at high humidity, *S. chartarum* is capable of producing mycotoxins on the respirator filter [34].

The effect of humidity on survivability of microorganisms on the filter material was confirmed by Maus *et al.* [36] who demonstrated that the viability of *Micrococcus luteus* and *Escherichia coli* declined within one hour after collection on ventilation filter media when no nutrients were present and the test was performed at low relative humidity (30%–60%). The study also suggests that bacteria cannot generally use modern filter materials as nutrients. For surviving, the bacteria use the more organic substances contained in the filtered air, coming from the user of the filtering device.

Wang *et al.* [33] showed that bacteria are unable to strongly colonize polypropylene respirator filter materials. They tested the survival and potential growth of *Bacillus subtilis* and *Pseudomonas fluorescens*. The bacteria were aerosolized and loaded on the respirator filters under three nutritional conditions: water, saliva, and TSB with 0–13 days of incubation. The authors showed that *P. fluorescens* cells couldn’t survive for more than three days on the respirator filter, while *B. subtilis* spores can remain viable for more than thirteen days. 

Brosseau *et al.* [31] investigated the survivability of microorganisms on eighteen types of respirator filters and five types of surgical masks stored at RH 85%. They showed that culturable cells could be recovered from filters following a five-day storage period. In their study, *B. subtilis* subsp. *niger* and *Staphylococcus epidermidis* showed high survivability and *Mycobacterium abscessus* demonstrated lower survivability.

In our study, the survival rate after five days was the lowest for *E. coli* (0%–0.1%) (depending on the moisture content) and *A. niger* (46%–80%). The highest survival rate was observed for bacteria *S. aureus* (2083-15,796%). As for other microorganisms, the rate ranged from 483% to 1632% (Figure 5f). The results show that if a worker exposed to the tested microorganisms uses the FFR, they may grow on the filter material and be harmful to the user’s health.

Brosseau *et al.* [39] suggest that policies regarding reuse, handling, and disposal of respirators and surgical masks should be carefully considered. If used respirators are stored in plastic bags, the humidity and nutrients collected on the filter material may favor microbial growth or survival [33].

Active microorganism forms, capable to growth on FFRP during usage and also microbial fragments and unculturable microorganisms in the laboratory conditions (VBNC) can be collected from the filter materials. Therefore, it is important to study the whole microorganism population directly from the material sample. In this way we can assess the biodiversity of used FFRP at workplaces.

Johnson *et al.* [35] studied the phenomenon of migration of microorganisms from the outer and inner layers of the FFR. They found that the reuse of respirators is burdened with a high risk of contamination of all their elements. Microorganisms can spread on the user’s skin, especially during handling and storage of equipment.

The results obtained by breathing simulation presented in this paper indicate that biocides that exhibit antimicrobial efficacy even at a moisture content of 40% should be used in reusable FFRs, practically from the very start of their use. 

## 4. Conclusions

The simulation of breathing shows that moisture content and temperature after 7 min of usage increased to 84%–92% and 29–30 °C, respectively. This condition is maintained as long as the FFR is used. This points to the fact that even with short-term use of the FFR favorable conditions for rapid growth of microorganisms deposited on the filter material can occur. The survivability of microorganisms on the filter material depends on the microorganism species and moisture content. At mass humidity ranging from 40% to 200%, the filter material is an appropriate environment for increase of survivability of *S. aureus* and *B. subtilis*, and the yeast *C. albicans* for five days. The environment, however, is not favorable for the development of *E. coli* and mold *A. niger*. This indicates that in workplaces where FFRs are used to protect workers against bioaerosols is necessary to develop and comply with appropriate rules for limited re-use and storage of FFRs, such as those defined in the guidance for healthcare institutions to protect health care workers from risks of exposure to infectious respiratory illnesses [52]. It is particularly important to store FFRs in a cool dry place in the intervals between each use and to maintain hygienic requirements when donning and performing a user seal check of previously used FFRs. It is also worth mentioning that one of the promising new directions of research related to the problem of microbial growth in the FFR is the use of filter materials containing bioactive agent in the construction of filtering equipment for protection of respiratory track against bioaerosols.
